# Four Methods for Monitoring SARS-CoV-2 and Influenza A Virus Activity in Schools

**DOI:** 10.1001/jamanetworkopen.2023.46329

**Published:** 2023-12-05

**Authors:** Jonathan Temte, Maureen Goss, Shari Barlow, David H. O’Connor, Shelby L. O’Connor, Mitchell D. Ramuta, Amra Uzicanin

**Affiliations:** 1 Department of Family Medicine and Community Health, University of Wisconsin School of Medicine and Public Health, Madison; 2 Department of Pathology and Laboratory Medicine, University of Wisconsin School of Medicine and Public Health, Madison; 3 Centers for Disease Control and Prevention, Atlanta, Georgia

## Abstract

This cross-sectional study describes 4 parallel approaches used simultaneously to monitor influenza A virus and SARS-CoV-2 activity within a Wisconsin school district during the Fall 2022 semester and briefly following winter break.

## Introduction

As community-based SARS-CoV-2 testing programs waned, alternatives for monitoring virus activity emerged. Kindergarten through 12th grade (K-12) schools provided excellent venues for surveillance given their role in respiratory virus amplification.^1^ Methods recently evaluated in school settings include rapid antigen testing (RAT), pooled specimen testing by reverse transcription–polymerase chain reaction (RT-PCR), wastewater monitoring,^2^ and screening with dogs.^3^ In this study we describe 4 parallel approaches (home-based specimen collection using RT-PCR, cause-specific absenteeism monitoring, school-based RAT, and air sampling) used simultaneously to monitor influenza A virus (IAV) and SARS-CoV-2 activity within a south-central Wisconsin school district during the Fall 2022 semester and briefly following winter break.

## Methods

The Oregon School District (OSD) serves 4114 K-12 students. We examined the concordance of 4 independent surveillance methods for IAV and SARS-CoV-2 in OSD schools, comparing the weekly results of each program in this cross-sectional study. See eMethods in Supplement 1 for detailed information. This study was approved by the University of Wisconsin Health Sciences institutional review board. ORCHARDS participants provided written informed consent; the other systems were exempted from informed consent by the institutional review board due to anonymity of composite data. We followed the STROBE reporting guideline.

The Oregon Child Absenteeism Due to Respiratory Disease Study (ORCHARDS) is a school-based respiratory virus surveillance study initiated in 2015; the methods have been described elsewhere.^1^ Specimens, collected with parental permission from students with acute respiratory infection, were tested for IAV, SARS-CoV-2, and other respiratory viruses using RT-PCR. Daily counts of cause-specific student absenteeism due to influenza-like illness (a-ILI) or COVID-19 (a-CoV) were collected from OSD as a component of ORCHARDS.^1^ Research staff provided training and Sofia 2 Flu+SARS Antigen Fluorescent Immunoassay RAT supplies (Quidel) to OSD health offices in August of 2021; rapid testing of students and staff has been ongoing during the academic year since that time.

Air samplers (ThermoFisher AerosolSense) were placed in communal gathering spaces (eg, cafeterias) in all 7 OSD schools, and cartridges were analyzed twice weekly for the presence of IAV and SARS-CoV-2 using a workflow as previously described.^4^ IAV and SARS-CoV-2 genetic material captured in air samples were detected using previously developed quantitative RT-PCR assays targeting the IAV M gene,^5^ SARS-CoV-2 N1 and N2, and RNaseP as an internal control.^6^ Two-sided *P* < .05 was considered statistically significant. Statistical analysis was performed from September 1, 2022, through January 28, 2023, using Cross Correlation Function version 1.0.10 in Free Statistics Software version 1.2.1 (Office for Research Development and Education, Wessa P).

## Results

From September 1, 2022, through January 28, 2023, 334 K-12 children participated in ORCHARDS, resulting in 114 IAV detections (34.1%) and 32 SARS-CoV-2 RT-PCR detections (9.6%). Student absenteeism monitoring recorded 1425 a-ILI days and 883 a-CoV days. Of 200 RAT contemporaneously performed in school nurses’ offices, 24 (12.0%) were positive for IAV and 9 (4.5%) for SARS-CoV-2. Air samples were positive for IAV in 43 of 154 school weeks and 12 of 22 weeks; and air samples were positive for SARS-CoV-2 in 101 of 154 school weeks and 22 of 22 weeks.

Influenza activity exhibited a peak observed in all 4 surveillance platforms from December 11 to 24, 2022, and dropped precipitously following winter break (Figure, A-D). SARS-CoV-2 was detected via air surveillance in at least 2 schools each week throughout the study period. SARS-CoV-2 was also detected continuously at moderate to low levels in both ORCHARDS-based RT-PCR and school-based RAT (Figure, E-H). Cross-correlations among the platforms were significant and without lags for IAV (Table).

**Figure.**
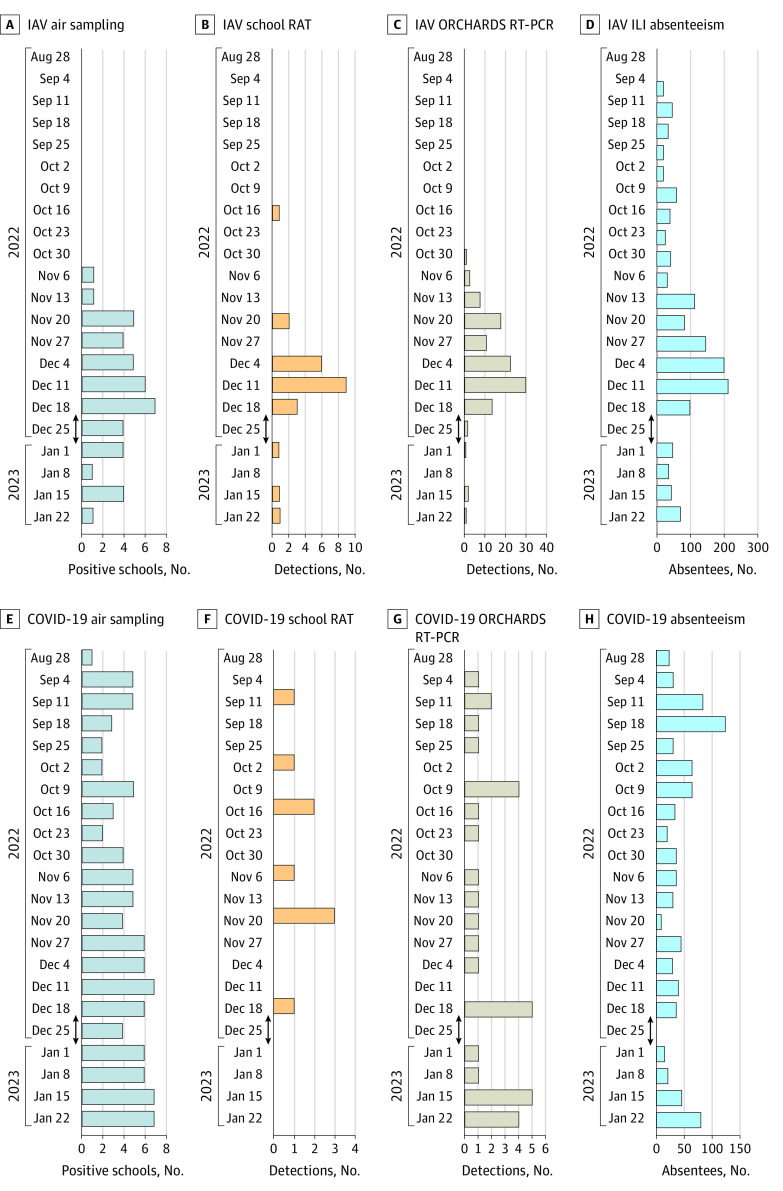
Comparisons of In-School Disease Surveillance Methods A-D, Influenza A virus (IAV) activity in the 7 K-12 schools in the Oregon School District, Dane County, Wisconsin, over 22 weeks between September 1, 2022, and January 28, 2023, illustrated by number of schools with positive cartridges per week via air sampling (A), number of positive IAV specimens per week via school-based rapid antigen testing (RAT) (B) and students participating in the Oregon Child Absenteeism due to Respiratory Disease Study (ORCHARDS) tested by reverse transcriptase–polymerase chain reaction (RT-PCR) (C), and number of children absent per week due to influenza-like illness (ILI) (D). Winter break occurred from December 22, 2022, through January 2, 2023, as depicted by vertical arrows. E-H, SARS-CoV-2 activity in these schools over the same period, illustrated by number of schools with positive cartridges per week via air sampling (E), number of positive SARS-CoV-2 specimens per week via school-based RAT (F) and students tested by ORCHARD RT-PCR (G), and number of children absent per week due to COVID-19 (H).

**Table.** Comparisons of Surveillance Platforms Showing Maximal Cross-Correlations With Associated Lags for Influenza A Virus and SARS-CoV-2 Detections per Week in the Oregon School District, Dane County, Wisconsin, Between September 1, 2022, and January 28, 2023

Surveillance platform	Cross-correlations for data series maximal correlation (lag, wk)^a^		
	Air sampling^b^	RAT	RT-PCR
**Influenza A**			
School-based RAT (n = 200; median [range] age, 15 [5-65] y)^c^	0.681 (0)	NA	NA
ORCHARDS respiratory samples tested by RT-PCR (n = 334; median [range] age, 12 [4-18] y;)^d^	0.823 (1)	0.891 (0)	NA
Influenza-like illness absenteeism	0.860 (1)	0.814 (0)	0.908 (0)
**SARS-CoV-2**			
School-based RAT^c^	−0.424 (−7)	NA	NA
ORCHARDS respiratory samples tested by RT-PCR^d^	0.494 (0)	0.503 (−4)	NA
COVID-19 absenteeism	−0.625 (2)	0.446 (9)	0.319 (0)

Abbreviations: NA, not applicable; ORCHARDS, Oregon Child Absenteeism Due to Respiratory Disease Study; RAT, rapid antigen testing; RT-PCR, reverse transcriptase–polymerase chain reaction.

^a^ Demographics of tested individuals by RAT and RT-PCR are provided. The bounds for significance at *P* < .05 were ±0.41. Minimal lags were noted for all IAV comparisons, but not for all SARS-CoV-2 comparisons. Cross-correlation calculated using Cross Correlation Function version 1.0.10 in Free Statistics Software version 1.2.1 (Office for Research Development and Education, Wessa P).

^b^ ThermoFisher AerosolSense cartridges were analyzed after elution and nucleic acid extraction using quantitative RT-PCR assays targeting the IAV M gene, SARS-CoV-2 N1 and N2, and RNaseP.

^c^ Nasal swab specimens were collected by school health office staff and tested immediately using Sofia 2 Flu+SARS Antigen Fluorescent Immunoassay.

^d^ ORCHARDS nasal swab specimens, self-collected at home, were tested for IAV and SARS-CoV-2 using a commercial multiplex assay kit based on RT-PCR for detection of SARS-CoV-2, IAV, and influenza B.^1^

## Discussion

Air sampling provided equivalent results to 3 parallel methods for SARS-CoV-2 and IAV monitoring using study participant sampling with RT-PCR, cause-specific absenteeism, and school-based rapid antigen detection over a 22-week period. Differing patterns between these 2 viruses emerged. While SARS-CoV-2 detections were stable (endemic), IAV demonstrated a substantial (epidemic) increase throughout November and December, receding after winter break. These contrasting activity patterns were reflected in each surveillance platform, except for RAT postwinter break, which did not detect SARS-CoV-2 activity. This study was limited by evaluating a single school district for only 22 weeks. Use of complementary surveillance tools in K-12 schools, including air sampling, may enhance detection of respiratory virus outbreaks.

## Notes

Supplement 1. **eMethods.** Methods Supplement

Supplement 2.
Data Sharing Statement
