# Supplementary material for: Four Methods for Monitoring SARS-CoV-2 and Influenza A Virus Activity in Schools
Source: JAMA Netw Open. 2023 Dec 5;6(12):e2346329. doi: 10.1001/jamanetworkopen.2023.46329 (PMC10698613; doi:10.1001/jamanetworkopen.2023.46329)
Supplement: Supplement 1. — eMethods. Methods Supplement [file jamanetwopen-e2346329-s001.pdf]

Supplemental Online Content

Temte J, Goss M, Barlow S, et al. Four methods for monitoring SARS-CoV-2 and influenza A virus activity in schools. *JAMA Netw Open.* 2023;6(12):e2346329. doi:10.1001/jamanetworkopen.2023.46329

eMethods. Methods Supplement

This supplemental material has been provided by the authors to give readers additional information about their work.

© 2023 Temte J et al. *JAMA Network Open.*

eMethods. Methods Supplement

Study design: This was a cross-sectional study. We have followed the STROBE guidelines in reporting.

Setting: The study was conducted within the Oregon School District (OSD), located in the villages of Oregon and Brooklyn, in Southcentral Wisconsin during the Fall 2022 semester, and briefly following winter break. Specific dates were August 28, 2022, through January 28, 2023, with the academic year starting on September 1, 2023. The OSD comprises three elementary (grades K-4), one combined elementary-intermediate (K-6), one intermediate (5-6), one middle (7-8), and one high school (9-12), and serves 4,114 kindergarten through 12th grade (K-12) students.

Participants: Participation is segregated by the surveillance platform:

1. ORCHARDS (ORegon CHild Absenteeism due to Respiratory Disease Study) is a school-based respiratory virus surveillance study initiated in 2015; the rationale and methodology have has been fully described elsewhere.[1] School-aged children (aged 4—18 years) from the OSD are eligible to participate if they report an acute respiratory infection, defined as having two of five respiratory symptoms (fever, cough, runny nose, nasal congestion, sore throat) with onset within seven days, and meet study criteria. ORCHARDS research staff obtains informed consent/ascent. During the study period, nasal swab specimens were self-collected, placed into viral transport medium, and transported to the Wisconsin State Laboratory of Hygiene for testing using RT-PCR for influenza A (IAV) and SARS-CoV-2 and for other respiratory viruses using respiratory pathogen panel. The University of Wisconsin Health Sciences Institutional Review Board has approved ORCHARDS.

© 2023 Temte J et al. *JAMA Network Open.*

2. ORCHARDS has been collecting and analyzing daily OSD cause-specific student absenteeism data since September 2014. Parents are required to call into the OSD automated absenteeism system when children are absent for any reason and provide additional information. Attendance staff enter the type of absence into Infinite Campus, a school information system. Four types of absenteeism data are routinely collected (total absenteeism, absenteeism due to illness, absenteeism due to an influenza-like illness, and absenteeism due to COVID-19). Data are aggregated by grade level and school and automatically sent to the ORCHARDS team using a secure file transfer protocol. For this study, we are using absenteeism due to an influenza-like illness (a-ILI) and absenteeism due to COVID-19 (a-CoV); a-ILI is defined as an absence due to illness with fever and at least one other respiratory symptom (cough, nasal discharge, nasal congestion, sore throat); a-CoV is defined as an absence due to COVID-19 illness or due to COVID-19 exposure for which the child is under quarantine. All data are de-identified, anonymous, and provided only as composite counts by school and grade level. Because of this, the absenteeism component of ORCHARDS has been deemed exempt by the University of Wisconsin Health Sciences Institutional Review Board and the Social Sciences and Educational Institutional Review Board.

3. The ORCHARDS team facilitated acquisition of rapid testing analyzers and supplies for the OSD and provided training on the use of Sofia 2 Flu+SARS Antigen Fluorescent Immunoassay (FIA) (www.quidel.com/immunoassays/sofia-2-flu-sars-antigen-fia) to OSD health office staff in August of 2021. Rapid testing of students and staff, however, has been under the purview of the OSD. This activity has been ongoing during the academic year since August 2021. The OSD obtains parental consent for testing at registration (beginning of the school year) for students. The

© 2023 Temte J et al. *JAMA Network Open.*

health offices test consented students presenting the health office with an acute illness. The health office also tests interested faculty/staff. This activity is non-research.

4. Working with the OSD, we placed ThermoFisher AerosolSense samplers in communal gathering spaces (e.g., cafeterias) of all seven OSD schools. Cartridges from each sampler were analyzed twice weekly for the presence of IAV and SARS-CoV-2 using a workflow as previously described.[4] Air samples were transported to the laboratory for nucleic acid extraction. IAV and SARS-CoV-2 genetic material captured in air samples were detected using previously developed quantitative RT-PCR assays targeting the IAV M gene[5], SARS-CoV-2 N1 and N2, and RNaseP as an internal control.[6] Air sampling has been deemed exempt by the University of Wisconsin Health Sciences Institutional Review Board.

Outcome Variables:

1. ORCHARDS (ORegon CHild Absenteeism due to Respiratory Disease Study) RT-PCR: number of positive RT-PCR detections of IAV and SARS-CoV-2 in each of 22 study weeks.
2. ORCHARDS Absenteeism: number of a-ILI and a-CoV occurrences in each of 22 study weeks.
3. OSD health office rapid antigen testing (RAT): number of positive RAT detections of IAV and SARS-CoV-2 in each of 22 study weeks.
4. Air surveillance: number of schools with a positive RT-PCR detection of IAV and SARS-CoV-2 in each of 22 study weeks.

Analysis: We used cross correlation analyses to evaluate the maximal correlation between surveillance platforms and to identify and lag or lead. We did not have any missing data. During one week of winter vacation, however, no absenteeism and no health office visits occurred as children/staff were not

© 2023 Temte J et al. *JAMA Network Open.*

present. These were therefore coded as 0 detections. We did not have any planned follow-up with any participant so there was no loss of participants to follow-up.
