# Supplementary material for: Four Methods for Monitoring SARS-CoV-2 and Influenza A Virus Activity in Schools
Source: JAMA Netw Open. 2023 Dec 5;6(12):e2346329. doi: 10.1001/jamanetworkopen.2023.46329 (PMC10698613; doi:10.1001/jamanetworkopen.2023.46329)
Supplement: Supplement 2. — Data Sharing Statement [file jamanetwopen-e2346329-s002.pdf]

Data Sharing Statement

Temte. Four Methods for Monitoring SARS-CoV-2 and Influenza A Virus Activity in Schools.
JAMA Netw Open. Published December 05, 2023. doi:10.1001/jamanetworkopen.2023.46329

Data
Data available: Yes
Data types: Deidentified participant data, Data (not involving human participants), Data dictionary
How to access data: jon.temte@fammed.wisc.edu
When available: With publication

Supporting Documents
Document types: None

Additional Information
Who can access the data: researchers whose proposed use of the data has been approved
Types of analyses: for any purpose
Mechanisms of data availability: with a signed data access agreement
